# Biopolitics at the Nexus of Chronic and Infectious Diseases

**DOI:** 10.1007/s11673-024-10405-4

**Published:** 2025-02-06

**Authors:** N. D. Brantly

**Affiliations:** Government & International Affairs (GIA), School of Public and International Affairs, Virginia Polytechnic Institute and State University, 223 Major Williams Hall, 220 Stanger Street, Blacksburg, VA 24061 United States

**Keywords:** Biopolitics, Global health, Risk, Diabetes, COVID-19

## Abstract

Non-communicable (chronic) and communicable (infectious) diseases constitute the leading causes of death worldwide. They appear to impact populations in developed and developing nations differently with changing trends in the landscape of human conditions. Greater understanding of changing disease burdens should influence the planning of health programmes, the implementation of related interventions, and policymaking efforts on a national and global scale. However, the knowledge of disease burdens does not reflect how states and global health organizations prioritize their efforts in addressing them. This work aims to address the discrepancy in public health priority setting by improving our understanding of how the two disease categories impact the human condition. It reviews two case studies, COVID-19 and type 2 diabetes, as representative cases of an infectious and a chronic disease, respectively, to answer the following question. How does biopolitics, as the governance of human bodies, at the nexus of infectious and chronic disease, impact national and global public health priorities? This work contextualizes and reframes the relationship towards disease categories by focusing on three primary themes: risk, current public health interventions, and funding priorities for each case study analysed. It argues that the politics over life at the nexus of chronic and infectious diseases, best conceived as future-oriented economic optimization, directs the efforts of prioritization in healthcare based on risk and responsibility-based relationship between multiple stakeholders.

## Introduction

Health is a valuable commodity for individuals and nations alike. Nations seek to have healthy and productive populations to ensure economic growth, increase workforce efficiency, and strengthen both human and national security (Elbe 2010). Individuals seek health to attain longer and more fulfilling lives or to avoid suffering. In contemporary society, efforts are made with advanced technologies and systems to surveil, regulate, control, and automate many aspects of human health for improved health outcomes, elevated economic growth, enhanced political stability, and increased global health security. These efforts appear to be forward-leaning endeavours based on perceived risks and threats to human health. Such efforts are viewed as progress based on neutral, objective, and evidence-based decision-making. However, they are rooted in the ways we perceive and understand human disease, which might be skewed, unbalanced, and unfair. A significant part of this understanding involves how we perceive communicable (infectious) and non-communicable (chronic) diseases.

Infectious diseases result from an infection by an illness-causing pathogen that can be transmitted among people through a variety of means. Chronic diseases have a prolonged course of illness and are often associated with poor lifestyle choices, use of harmful substances, advanced age, and genetic or environmental factors. Chronic and infectious diseases constitute the leading causes of death around the world, with changing trends in the landscape of human conditions (WHO 2024b). The prevalence of multiple chronic conditions is increasing globally (WHO 2023b) and especially in developing nations (NIH 2023). The increasing burden of chronic disease is expected to “cause three-quarters of the disease burden in low- and middle-income countries by 2030” (NIH 2023). This estimation of the future state of global health is concerning, yet even today, public health and funding priorities are lagging and do not reflect the realities and current changes in the landscape of human conditions (Ballreich et al. 2021; IHME 2023).

Chronic conditions constitute the leading causes of death in the United States, with heart disease, cancer, stroke, diabetes, and kidney disease among them (Kochanek et al. 2024). COVID-19 emerged as the third leading cause of death in 2020, contributing to a decrease in life expectancy and an increase in mortality (Murphy et al. 2021). In 2022, COVID-19 was reported as the fourth leading cause of death, and in 2023, it provisionally became the tenth leading cause (Ahmad, Cisewski, and Anderson 2024). Shifting patterns of mortality indicate the effects of the COVID-19 pandemic on the mortality burden and disruptions in healthcare services that might have hampered early detection and chronic disease management (Ahmad and Anderson 2021). According to the 2023 provisional mortality report, non-communicable diseases are responsible for most deaths in the United States (Ahmad, Cisewski, and Anderson 2024). The unfortunate state of health in the United States, as evidenced by increasing mortality and prevalence of disease, is further exacerbated by high costs, inequitable access to healthcare services, social determinants of health, and the health literacy gap.

When looking at the same information globally, we can see a similar picture. While infectious diseases constitute the majority of deaths in low-income countries, the prevalence of deaths from chronic conditions increases along with gross national income (WHO 2024b). In fact, the World Health Organization (WHO) has identified diabetes as the rising cause of death in lower-middle-income countries, while the presence of diabetes among the top ten causes of death for middle- and high-income countries shows a worrisome trajectory (WHO 2023a). An understanding of causes of human mortality and of patterns and trends in disease prevalence does not appear to inform our efforts to improve how people live, both in the United States and globally. Some researchers highlight positive changes in the financial support and recondition of non-communicable diseases (NCDs), such as diabetes, yet prioritization in global health remains fragmented and inequitable with seemingly no coherent global health agenda (Smith and Gorantla 2021).

“It is important to know why people die to improve how people live” (WHO 2024b). This is a significant statement, as understanding human mortality is a powerful tool in identifying public health vulnerabilities and priorities, allocating resources, and directing state focus toward activities that will have the greatest benefit for the population. Yet, there is a large discrepancy in the prioritization of population health issues. NCDs receive less funding than communicable diseases, even though they are responsible for a greater percentage of deaths per year in the United States and globally. As global health scholar Jeremy Youde writes, “[t]he global health governance system is overwhelmingly geared towards infectious disease, even though the vast majority of deaths worldwide are due to NCDs like cancer, heart disease, and respiratory conditions” (2018). Some researchers link increased funding and increased awareness pertaining to a health condition to the reduction of mortality and improved patient survival (Hall et al. 2020). The mortality data in the United States and globally do not accurately reflect how states decide to approach threats to human health. What logics are driving national and global health policies, then?

In answering this question, the aims of this article are twofold. First, it aims to improve our understanding of diseases and their impact on the human condition. Second, it aims to address the discrepancy in global health priority setting by analysing the perceptions of the two disease categories through the lens of biopolitics. It reviews two case studies, coronavirus disease 2019 (SARS-CoV-2 or COVID-19) and type 2 diabetes (T2D), as representative cases of an infectious and a chronic disease respectively, to answer the following question. Focusing on the nexus of infectious and chronic diseases, how does biopolitics, as the governance of human bodies, impact national and global public health priorities? This work contextualizes and reframes the relationship towards disease categories by focusing on three primary themes: risk, current public health interventions, and funding priorities. The first one addresses the perceived and real risks and threats brought about by the health condition discussed. The second one, public health interventions, reviews current efforts in addressing the health condition. The third one, funding priorities, includes the analysis of national and local funding priorities in select nations to highlight possible trends pertaining to each case study. This work argues that the politics over life at the nexus of chronic and infectious diseases, best conceived as future-oriented economic optimization, directs the efforts of prioritization in healthcare based on risk and responsibility-based relationships between multiple stakeholders.

This article proceeds in four sections, starting with a discussion of the concept of biopolitics and how it is applied to disease considerations. The second section addresses the case studies through the analysis of each focus area as outlined above. These focus areas emerged as primary themes from the literature review and are used to highlight the differences between the two disease categories that might explain the discrepancy in healthcare prioritization efforts. The third section draws on findings from the case study analysis to create a framework within which to contextualize and then reframe the relationship toward disease categories. The framework review provides insights about the politics over life across levels (individual versus population) and across focus areas to help explain the discrepancies in national and global health priority setting. Finally, the paper concludes with a summary of findings offering insights into further research.

## Biopolitics in Disease Considerations

COVID-19 and diabetes, while very different in terms of disease onset, are both referred to as pandemics by regulatory experts (WHO 2024a; Singer et al. 2022) and in popular media discourses (Khan 2021; Abbott 2022; NYT 2024). Calling a health condition a pandemic is a form of discipline as it appeals to the moral responsibility of individuals to strive for, achieve, and maintain a disease-free state for the security of self and others. Part of the disciplinary process includes emphasizing the urgency and extremity of the condition to urge a return to a “normal” disease-free state (Guthman 2009). Normalizing and moralizing discourses work to discipline and govern individual citizens through regulated choices and aspirations that take the form of individual empowerment and freedom toward self-fulfilment, self-control, and discipline. In addition to being a moral problem, disease can be framed as a social problem (a matter of the environment) or a medical problem (a purview of regulatory experts to be approached within a clinical setting).

The mortality information communicated by the national and global health organizations is vital in demonstrating the dangers to human health caused by chronic and infectious diseases. The heightened sense of urgency, spurred by pandemic discourses and mortality data, prompts individual and collective response. However, people are no longer governed simply as “political subjects of law” but also as living beings who form units or populations, both small (local sub-populations) and large (global population), with average life expectancy, mortality rate, morbidity rate, and other metrics to act as “obedient biopolitical subjects” (Lorenzini 2021). The lives and health of people across levels become a matter of political decision-making. Therefore, disease and the discrepancies in national and global health priority setting can also be framed as a biopolitical problem. Biopolitics is a concept that focuses on the ways in which human life and biological processes are managed, regulated, and influenced by political powers. The French philosopher Michel Foucault popularized the concept in the late twentieth century by exploring how states exert control over populations through institutions, laws, and norms that regulate life, health, and bodies (Foucault 2008). Pandemic discourses, mortality data, and other health statistics not only frame health issues and calls to action but also influence individual choice and the regulation of different aspects of life to ensure greater security and better health outcomes for all. If the above is true, and both COVID-19 and diabetes are addressed with a heightened sense of urgency, why then is there such a stark difference in the way these two conditions are addressed? A better understanding of biopolitics in disease considerations can help explain the difference.

Nikolas Rose calls the vital politics—the interest of the state in the lives of those who are governed—of the eighteenth and nineteenth centuries as the politics of health, while he views the vital politics of the current century as the politics of “life itself” (Rose 2007a). The difference is that while in the past the state was primarily concerned with the rates of birth, death, and disease and with the water quality, sewage, and the lives of those living in higher-density areas, today it is concerned with the “growing capacities to control, manage, engineer, reshape, and modulate the very vital capacities of human beings as living creatures” (Rose 2007a). The focus of the government, more so today than ever before, has shifted towards the control and oversight of the population and the population’s health under the purview of promises for improved biosecurity and better health outcomes. Chronic and infectious diseases pose a significant risk not only to human health (biological security) but also to the flow of goods and services in and across nations (economic security), to the maintenance and the improvement of the lifestyle we enjoy today (national security), as well as to the political apparatus itself (political security).

Biopolitics, as the governance of human bodies and human societies (Hinchliffe et al. 2016), is changing in several ways with efforts to control, manage, and reshape individuals through technological means. For example, the World Health Organization (WHO) promotes innovation, investment in data, health information systems, and digital solutions to obtain timely, reliable, and actionable data. The WHO goes so far as to say: “With good data we can ensure no one dies from a preventable, treatable illness. With good data we can reach vulnerable communities. With good data we can achieve the SDGs [Sustainable Development Goals] and leave no one behind” (WHO 2023c). The utility of modern technologies and data for national and global health cannot be dismissed. However, the quote above points to problems with using data as the sole solution to preventable and treatable diseases such as COVID-19 and T2D without consideration for other important factors, such as lifestyle or the social determinants of health, to name just a few. Such heavy reliance and trust in technological solutions and data make one believe that the “ideal” state of health, as something of value, can be captured and easily maintained with good data alone.

Reliance on technological solutions is characteristic of biopolitics with efforts to understand, manipulate, control, and optimize life through the use of technologies and the “economies of vitality,” where the research of biological value, cures, health optimization, and scientific truths is closely linked to capital and shareholder value (Rose 2007a). The biopolitics of disease is changing the relationship between individuals and those in positions of authority, as well as the relationship among individuals and the perception of life. Biopolitics itself, as a “political rationality which takes the administration of life and populations as its subject” (Adams 2017) “to ensure, sustain, and multiply life, to put this life in order”(Foucault 1984) has transformed to become “inextricably intertwined with bioeconomics” (Rose 2007a). The bioeconomy, often represented in rates and numbers, entails novel forms of alliances between political authorities and promissory capitalism, investment, and allocation of capital according to promised future returns (Rose 2007b). Such an alliance reflects the connection between health and wealth that guides both national and global resource allocation.

Public perception of health and disease is often inspired by profit-driven corporations that encourage active participation in the consumption of medical services, pharmaceuticals, and biomedical technologies. Corporate influence is further intertwined with scientific research, the practice of medicine, and the delivery of healthcare services, leading to the blurring of the boundaries between industry and academic and public health interests (Hunt et al. 2021). Public perception of disease is thus skewed due to pervasive industry involvement in biosecurity, biomedicalization, and interventions aimed at the development of markets for profitable pharmaceuticals (Clarke et al. 2010). Commercial interests align with the state’s interest in overseeing, managing, and influencing the population through modern technologies. State interests align with commercial interests as exemplified by BARDA (Biomedical Advanced Research and Development Authority), established under the U.S. Department of Health and Human Services to help address public health emergencies (HHS 2024). One way to explain this is to acknowledge that we live in a risk society that views the risk of future disease, illness susceptibility, and potential for adverse health outcomes as requiring additional regulation, discipline, and interventions (Lupton 1999). Communicable and non-communicable diseases are closely linked to risk, but chronic diseases like diabetes are often framed as progressive, carrying a significant threat for patients with ever-present short and long-term risks becoming increasingly egregious over time (Brantly 2023). Infectious diseases like COVID-19, by contrast, are often framed as acute, urgent, and a matter of emergency and security (Colebrook 2023). These perceptions of risk influence our understanding of disease and the difference in approaches to addressing illness, all in the name of better health outcomes.

Some scholars have argued that quantification of health, like the Disability Adjusted Life Year (DALY) metric, reimagines health as a site of investment, as a form of human capital, and as an economic project focused on a hypothetical and imagined future (Kenny 2015). Quantification of health also creates a new form of biopolitical technology of power that is shaping contemporary national and global health governance. Here, technology is not only devices, instruments, and equipment but also bodies, spaces, relations, knowledge, and systems of judgement. Using the logic of health quantification, vital components of life are dismantled and reassembled as potential investments and revenue streams (Kenny 2015). Individuals also align with corporations and states in the prevention of risk and, in so doing, also become economic agents engaged in the production, consumption, investment, and exchange of goods (knowledge, data, biovalue) and services (self-care, home care, education, communication, testing). Individuals, as economic agents, are working to improve health today to ensure a future return on investment in the form of greater productivity and a revenue source. They become future-oriented agents of the self, focused on self-optimization. Health becomes a risk-minimizing effort and a practice of investing in one’s health to ensure an “ideal” state of health in the future. Individual self-optimization efforts are guided by population-level norms and standards established under the influence of commercial interests and regulatory experts.

The balancing of individuals as economic agents with the goals of the state and industry to ensure the well-being of the population is achieved by focusing on self-optimization through the market, thereby shifting the responsibility and the prioritization of individual self-improvement over welfare state provision. Kenny demonstrated how health systems are designed according to the logic of economic optimization. This optimization is focused on the imagined future that becomes an integral feature of the present. The future is imagined as a disease-free society with an “ideal” state of health that can be achieved through diligent action in the present. It is a form of anticipatory bioethics where the future determines the present (Camporesi 2017). The future is conceived based on the considerations of risk, both real and perceived. It determines the efforts of health optimization today via technological innovation, data, and the assignment of responsibility to individuals, states, and other stakeholders. As a result, efforts of prioritization in healthcare cannot be divorced from commercial interests that often seek to address individual health needs by using population-level health data to create targeted products. The term “commercial determinants of health” is often used to refer to the activities of the private sector that impact public health, well-being, and health systems, either positively or negatively, while “enabling political economic systems and norms" (WHO 2024c). Since both infectious and chronic diseases call for public health interventions and state responses, the perceptions of risk, reliance on the market, and the assignment of individual responsibility for disease necessarily influence the current balance in national and global priority setting.

As Colebrook states, “public health measures are not geared towards the survival of individuals but the management of populations” (Colebrook 2023). Other scholars argue that industry increasingly influences federal regulatory bodies and dominates the production of medical knowledge and the setting of medical standards (Hunt et al. 2021). Either way, consideration for an individual’s specific health needs becomes secondary to data-driven decision-making and future-oriented risk reduction through increased use of pharmaceuticals and advanced technologies. The quantification of health using data and health metrics lends those in the position of authority objectivity, expertise, and legitimacy. Quantification can then be used to impart norms and standards to systematically influence human behaviour and health practices.

To summarize, the entangled nature of biopolitics and bioeconomy favours the growth of disease categories, the growth of regulatory expertise, and the growth of health commodities. It also favours greater population compliance through regulatory requirements and policies. It is reliant on hierarchies in the structures and values of lives, “producing and multiplying vulnerability as a means of governing people” (Lorenzini 2021). Those in the position of authority aim to intervene in the “vital characteristics of the human body and human existence” via regulatory controls to manage life “in the name of the well-being of the population” (Rose 2007a). The analysis of infectious and chronic diseases that follows reveals not only vulnerabilities but also inconsistencies in disease categories that are based on risk and responsibility-based relationships between multiple stakeholders. Although there are differences, both disease categories are governed by a similar biopolitical logic.

## Case Study Analysis: COVID-19 and Diabetes

The boundaries between disease categories—between non-communicable (chronic) and communicable (infectious) diseases—are not as clear as they might appear at first glance. For instance, while HIV (human immunodeficiency virus) is a virus that attacks one’s immune system, infected people experience both acute infection and chronic disease that will transition into AIDS (acquired immune deficiency syndrome) without treatment (CDC 2022b). Likewise, COVID-19 may transition into long COVID, a chronic illness requiring care for an extended period (CDC 2024). Diabetes is not infectious in a traditional sense, yet narratives indicate the “spread” of diabetes across communities globally (Hu 2011; Rabin 2022). COVID-19 and diabetes were selected as representative conditions for their prominence in media discourses, for their prevalence globally, and for the urgency with which both conditions are characterized as pandemics in need of immediate action. Diabetes and COVID-19 also interact synergistically, leading to an increased number of deaths from both (Lv et al. 2022; Summan et al. 2024). Many individuals with COVID-19 infection who experienced hospitalizations and serious health complications had diabetes (Fang, Karakiulakis, and Roth 2020). It is important to acknowledge this link within and among disease categories in the discussion of healthcare priority setting as it makes distinguishing between and attributing effects of illness or death more complicated.

On January 30, 2020, the WHO Director General declared that the outbreak of COVID-19 constituted a Public Health Emergency of International Concern (WHO 2020). The significance and urgency of the COVID-19 pandemic was quickly recognized by the international community. The COVID-19 pandemic has tested national health crisis preparedness, supply chains, and manufacturing resilience. It resulted in significant economic setbacks and hampered travel and tourism globally. Unlike COVID-19, diabetes has a long history dating back to 1552 BC (Kilgour 1993). Diabetes impacts a patient’s ability to self-regulate blood glucose, which is vital for human life. T2D—over 95 percent of all diabetes cases—is considered a lifestyle disease and is a major cause of lower limb amputations, blindness, kidney failure, and cardiovascular disease. It also is a significant risk factor for adverse outcomes in infectious disease comorbidity. Both conditions have detrimental impacts on U.S. and global economies and mortality rates, yet they are approached very differently in terms of national and international efforts.

### Risk Considerations

While COVID-19 is a novel virus, the discussions of an emerging new pathogen are not novel. National public health experts and the global health community had discussed the emergence of new pathogens, as well as national and global preparedness, for many years before COVID-19 made its appearance (Holloway et al. 2014). At the very core, these discussions are risk-based and aimed at easing the burdens of disease to human life and society. Emerging new pathogens or mutating known infectious agents create fear and speculation about the severity of the disease to come. Infectious diseases with pandemic potential are, therefore, an ongoing concern requiring proactive action to secure the best possible future with reduced risks to people and nation-states.

COVID-19 has real and perceived risks that are continually debated across all political levels. The real risks include the impact of disease on human health. They include the symptoms, health complications, hospitalization, and cost of testing and treatment. Additional risks include one’s inability to work and care for self and others, as well as the associated stress, anxiety, depression, and other mental health issues. They also include the impact of disease on one’s travel plans, one’s access to indoor and outdoor spaces, and one’s ability to infect others. The perceived risks are more challenging to define because they are future focused. They include the fear of the unknown, the uncertainty of the future impact of disease, possible mutations of the virus, and the long-term impact of the novel virus on one’s health, one’s well-being, and longevity. Additionally, perceived risks include the uncertain impact of infectious disease on the economy, society, and the government. The uncertainty prompts proactive steps and might be influenced by misunderstanding of real risks, by misinformation, or simply by the lack of knowledge.

Diabetes has a long history with rising rates of disease (CDC 2021b) and prediabetes (a condition of elevated risk factors for diabetes) in the United States (Sharpe 2020) with particularly worrisome trends among adolescents and young adults (Andes et al. 2020). Diabetes also has real and perceived risks. The real risks for diabetes include those highlighted for COVID-19 above, with some additional ones. These include the prolonged course of illness and lifelong disease management with associated high annual costs. Diabetes requires constant diligence to prevent irreversible complications and continuously degrading health. It can cause both short and long-term inability to work, chronic pain, discomfort, suffering, and life-long disability. Additionally, individuals with diabetes suffer from a wide range of psychiatric disorders, sleep disorders, eating disorders, and stress-related disorders at a higher prevalence than the general population (Brantly 2023). Diabetes also increases the risk of heart attack, stroke, foot ulcers, infection, limb amputation, blindness, and kidney failure (WHO 2023a) and elevates the risk of adverse effects from communicable diseases. There are fewer perceived risks for diabetes compared to COVID-19. These include genetic markers, family history of diabetes, ethnicity, and socioeconomic status that might necessitate frequent testing and the use of preventative pharmaceuticals. Perceived risks of diabetes are focused on the past, not the future. One’s ethnicity, family history, or genetic makeup cannot be changed over time. Perceived risks of diabetes also depend on the individual’s lifestyle and daily habits. While lifestyle and daily habits can change, it is up to the individual to change them. As a result, additional perceived risks of diabetes include individuals themselves, their behaviours, shortcomings, and their inability or unwillingness to follow prescribed regimens to achieve better health outcomes.

Both conditions have real risks that contribute to increased healthcare costs and suffering. Perceived risks for COVID-19 are future-focused. They seem to emerge from the outside of the body, from the infectious agent found in the environment. Diabetes perceived risks are focused on the past. Despite established social and environmental causes, perceived risks of diabetes seem to emerge from the inside of the body, from the genes or one’s poor choices. As a result, COVID-19 is perceived as a threat from the outside, necessitating a shared response to a common adversary. Diabetes, with a threat emerging within the individual, calls for personal responsibility and individual response. While COVID-19 appears to be non-discriminatory in how it affects others, everyone is at risk and in need of outside interference. Diabetes appears to affect individuals based on their choices or circumstances, so it requires vigilance and control from the self. Neither COVID-19 nor diabetes are social equalizers— quite the opposite; both expose how contemporary society depends structurally on the continuous production of differential vulnerability and varying levels of social disparities. However, perceptions about the risk and origin and expectations about these diseases might explain why approaches to address them differ so greatly.

### Public Health Interventions

Early interventions for COVID-19 included nonpharmaceutical interventions such as personal hygiene, isolation, quarantine, and personal protective equipment (PPE) use. These measures have been studied extensively and shown to be effective in the prevention of communicable disease transmission (Peak et al. 2017). COVID-19–related lockdowns, while effective at halting the rapid spread of disease, adversely affected the provision of outpatient services, early diagnosis, and preventive treatments for a full range of health conditions. As a result of the lockdowns and physical distancing requirements, the utilization of telehealth services has increased. Public health efforts during the pandemic were helpful in curtailing the spread of the virus, facilitating access to care, and advocating for increased innovation and cooperation to ensure public health security.

International efforts in testing and vaccination development have resulted in record-breaking outcomes. The COVID-19 vaccine, an effective way to prevent severe infection (CDC 2023b), had to be prioritized by healthcare providers and at-risk population groups before nations had enough for their populations. However, existing fears and anxiety regarding vaccine acceptance and hesitancy were further impacted by the misinformation, lowering the intent to accept a vaccine and threatening the goal of achieving herd immunity against COVID-19 (Loomba et al. 2021). Additionally, the COVID-19 pandemic has redirected public health efforts and resources from other relevant issues, as well as increased the use of digital technologies to increase population surveillance, case identification, and tracking in the name of improved preparedness (Budd et al. 2020). Current efforts to address COVID-19 include vaccinations, home self-testing, and quarantine. These are reactive efforts. Proactive approaches include education, the stockpiling of vaccines, PPE, and biomedical equipment. They also include efforts to enhance preparedness for future infectious disease outbreaks, such as incentivizing domestic manufacturing to expand the supply chain related to future pandemics (FEMA 2020).

In 2010, the National Diabetes Prevention Program (NDPP) was created to address the increasing burden of T2D and prediabetes in the United States (CDC 2022c). The NDPP is a public-private partnership of many organizations focused on lifestyle change programmes to address diet and exercise to reduce the risks associated with T2D (CDC 2022c). However, such lifestyle-change programmes are usually short-term (up to a year) and offered at a cost; some are offered online only, and none address the underlying societal issues that contribute to the increasing prevalence of diabetes and prediabetes globally (CDC 2023d). The CDC, the American Diabetes Association (ADA), public health departments, and a variety of organizations are working on educational efforts, awareness campaigns, and interventions to inform the population of prediabetes and the risks associated with diabetes, targeting populations from adolescents to the elderly. Despite the resources currently available for diabetes education and prevention, there is still inequitable access to resources and issues with affordability.

Most public health efforts targeting diabetes are reactive and involve already-diagnosed individuals or those with prediabetes. Reactive approaches utilize data and biomedical technologies to manage disease using pharmaceuticals. Proactive efforts are very limited and primarily include education. In the United States, there are currently no population-level public-policy interventions aimed at creating improved conditions for all individuals through increased taxation, a ban on fast-food sales, or urban re-planning that necessitates physical movement of people. This tracks with scholarship arguing that there is a preference to maintain the disease-promoting lifestyle by ingestion of pharmaceuticals over taking action to impose restrictions to effect change (Greene 2007). Some limited approaches have been adopted in countries such as Brazil, India, Ireland, the United Kingdom, and South Africa, which have focused on the taxation of sugar-sweetened beverages (Kruger et al. 2021). Diabetes can be characterized as a social problem, indicating the need to focus on population-level mitigation and prevention efforts. However, a contrary characterization is more often applied, with the emphasis placed on individual responsibility in diabetes discourses that are closely tied to obesity discourses (Guthman 2009).

Interestingly, diabetes discourses themselves can induce stress, anxiety, and mental health issues, impeding efforts to educate and encourage action of self-control and lifestyle change. Despite these challenges, individuals diagnosed with diabetes are often viewed as offenders since they do not follow established norms and expectations of self-care. Thus, public health approaches to diabetes are likely to focus on education and self-management. Individuals diagnosed or at risk of COVID-19 are often viewed as victims of an external environmental threat. Thus, approaches to COVID-19 require collective action and focus on greater public health interventions. COVID-19 is perceived as a collective enemy, similar to the invasion of a political adversary. It is a virus that calls for common responsibility and necessitates a joint response. As a result, no fault is ascribed to people who fall ill due to COVID-19, as the fault rests with the viral threat. Diabetes, by contrast, involves no such apparent invasion and is therefore considered to emerge from an innate flaw in the individuals themselves. In fact, those with diabetes are seen as a threat to the state because of their perceived irresponsibility, cost to the public, and fragile biological constitutions.

### Funding Priorities

With the emergence of COVID-19, the fear, hype, and significance of the emerging pathogen redirected the attention and funding of individuals, businesses, and governments towards a single adversary that endangered human health. The Centers for Disease Control and Prevention (CDC) was awarded a total of US$2 billion from the American Rescue Plan to bolster the governmental public health response to the COVID-19 pandemic and to establish, expand, and sustain a public health workforce (CDC 2021c). This is a significant increase in funding to support the hiring of personnel at the local health department or community-based organization level dedicated to COVID-19 efforts. In 2020, the CDC was awarded nearly $730 million in funding intended “to carry out surveillance, epidemiology, laboratory capacity, infection control, mitigation, communications, and other preparedness and response activities” (CDC 2023a). The funding received by the CDC for COVID-19 assistance and relief as of August 2021 amounted to over US$67 billion (CDC 2021a). USAspending.gov, which tracks federal awards, loans, and federal assistance, reported total COVID-19 spending in the amount of US$3.6 trillion across different U.S. agencies (2023).

The WHO worked on a global scale to “combat the COVID-19 pandemic” through the Strategic Preparedness and Response Plan (WHO 2021). Despite some funding gaps (WHO 2022), governments around the world took extensive action to reduce and limit the economic and human impact of the COVID-19 pandemic (IMF 2022). The pandemic was able to generate not only significant financial resources but also the unified action of a diverse group of stakeholders. Expenditures related to COVID-19 might be perceived as investments into risk-reduction efforts and improved future pandemic preparedness. This investment is expected to bring forth worthwhile results in the form of vaccines and other pharmaceuticals and biomedical technologies, as well as greater security, improved productivity, and less risk. Approaches to COVID-19 appear to spur commercial production that supports corporate profitability.

Diabetes is a costly condition for both individuals and healthcare systems. In addition to the high and continuously increasing costs of diabetes care in the United States (Riddle and Herman 2018) and the loss of productivity, individuals living with diabetes rely on affordable healthcare services and treatments, including insulin, for their survival (PAHO 2022). In September 2018, the CDC awarded US$45 million to state health departments of all fifty states and the District of Columbia under a new five-year cooperative agreement for T2D prevention and management efforts (CDC 2021d). Despite these efforts, many U.S. residents are living with a chronic condition, with nearly 50 per cent of individuals over the age of fifty-five having two or more chronic conditions (RFAH 2021). Additionally, diabetes is one of the largest global public health concerns, with negative effects on public health and socio-economic development around the world (Lin et al. 2020). It carried with it significant costs in terms of mortality and reduced life expectancy. The global costs of diabetes are also increasing, with the global economic burden of diabetes expected to increase from US$1.3 trillion in 2015 to US$2.5 trillion by 2030 (Bommer et al. 2018).

COVID-19 is associated with an acute onset of disease and a rapid impact on society with a high level of threat to national and global economies. Diabetes is associated with slow onset, slow impact on society, and a lower level of immediate threat to the economy. Chronic disease appears to be perceived as a choice driven by one’s ignorance towards societal standards set for a healthy lifestyle, including daily exercise (CDC 2022a) and healthy food consumption (USDA 2024). This ignorance is then coupled with the determination of deservingness. Diabetes is determined to be unworthy and undeserving of increased funding and other public resources because it might be considered malleable, flexible, and fixable with changes in one’s lifestyle, with increased awareness and education. On the other side, infectious disease is perceived as a fixed category beyond one’s control, requiring collective response via vaccination, travel restrictions, and work-from-home policies.

Despite the estimates that every dollar invested in proven non-communicable disease interventions in low- and lower-middle-income countries will generate at least seven dollars in “increased economic development or reduced health care costs by 2030” (CDC 2021e), expenditure related to diabetes are less likely to be viewed as a good investment. Proactive approaches to diabetes can be seen as long-term investments without short-term returns. It means giving up resources today for a potential chance to improve health in the future. Long-term investments are risky as they do not offer an immediate return on investment. It is easier to shift the responsibility to the individual to make better choices in the system designed to motivate them to do otherwise than to change the system itself. These calculations, in turn, shape the biopolitical decisions that determine whose lives are worth investing in and who, by contrast, should be starved of resources.

Both conditions are closely tied to commercial interests and commercial activities. While the focus on COVID-19 is aimed primarily at treatment and preparedness, the focus with diabetes is on disease management and education. The big business of diabetes management and the growing market of pharmaceuticals is chronologically related to specific screening, diagnosis, treatment, and diabetes management guidelines modifications (Hunt et al. 2021). It is more cost-effective to manage diabetes than to prevent or cure it. As a result, health becomes a form of human capital to be invested in, researched, and developed (Kenny 2015). It becomes a matter of commercial, scientific, and political reasoning and manipulation. Health becomes an economic project with pros and cons for investment, with returns and losses, as well as short- and long-term considerations.

## Framing and Reframing Life Politics

The case study analysis above demonstrates that despite the entangled nature of infectious and chronic diseases, there are differences in the way the two disease categories are perceived. The case studies reveal vulnerabilities and inconsistencies in the understanding of risk where future-focused external threats to human health take precedence over current internal threats. Public health priority-setting is inherently bioethical, not only because it involves crucial decisions about justice, equity, and values guiding health policies, but also because it has profound ethical implications for individuals and societies. Today’s efforts to prioritize at the national and global levels are closely linked to the way we understand human health and disease. The complexity of disease perception is reflected not only in the way it is communicated in popular discourses but also in the way the disease is approached through data, quantification, and increased use of technologies. The use of health metrics and technologies allows for the calculation and estimation of the state of health as promissory capital, promising future returns on the allocation of resources. Health becomes a goal of future-oriented economic optimizations. The allocation of resources likewise becomes a strategic approach based on the risk and responsibility-based relationship between different stakeholders.

The politics over life at the nexus of infectious and chronic diseases is shaped by commercial influences that are closely tied to scientific and political reasoning. To achieve health, one must reduce disease and associated risks. The perception of health as a form of human capital guides efforts in risk reduction, turning health into an object for manipulation and investment. It also shapes public health policies and resource allocation decisions. Biopolitics facilitates the shift of national and global public health priorities away from approaches based on current needs and suffering towards those focused on perceived risk and responsibility. This shift is evident in the considerations of risk, public health interventions, and funding priorities discussed above. Populations become the target of the state under the rationale of enhanced security, which is further exacerbated to the detriment of the welfare of the populace in times of conflict (Brantly and Brantly 2023). In peaceful times, biopolitics shapes the discrepancies in prioritization and justifies them in the name of greater security and better future health outcomes. It plays a central role in how different stakeholders prioritize time, effort, funds, and other resources to address health conditions. States may allocate a significant portion of their budget to infectious disease control programmes due to perceived immediate threats to public health and national security, as evidenced by the COVID-19 pandemic. Corporations driven by profits and perceived urgency may invest more in research and development of treatments and vaccines for infectious diseases as well as in pharmaceuticals to manage chronic diseases. Prioritization at various levels is influenced by the values and preferences emerging from perceptions of diseases, including future-oriented efforts to optimize life, self-improvement, and risk reduction.

The mortality data and other health statistics demonstrate the frame of suffering within the national and global populations. This data shows that chronic conditions dominate within this frame in the United States and increasingly globally. The case study analysis helps explain some of the reasons why the prioritization efforts represent a skewed picture of disease within the population. The biopolitics in disease considerations make it visible that the vision of the future state of health and disease within the population reflects the views of those in the position of authority. This vision is fuelled by biosecurity, biomedicalization, and bioeconomy considerations, as well as by the inequitable shift of responsibility for infectious and chronic disease among different stakeholders.

Table 1 below represents a generalized summary of the case study analysis and the literature review to highlight several points of variation between the two types of conditions reviewed. This table aims to highlight the differences in real and perceived risk perceptions, the difference in public health efforts (reactive and proactive), and the difference in responsibility assignment for disease (offenders and victims). These are important to our understanding of why infectious conditions might result in a shared responsibility for an outbreak while chronic conditions, such as diabetes, result in an individual responsibility of the patient for their health, life choices, and health outcomes. The disparity in prioritization efforts is not only about disease onset (acute or slow) but also about the perceived impact of disease on society and the economic and political well-being of nations, which is intimately intertwined with commercial interests that influence perceived risks and responsibilities for diseases and their outcomes.

**Table 1:** Summary of Perceptions at the Nexus of Chronic and Infectious Diseases

**Infectious Disease** **(COVID-19)**	**Chronic Disease** **(Diabetes)**
Acute Onset	Slow Onset
Quick Impact on Society	Slow Impact on Society
Shared Common Responsibility	Personal Responsibility of Patient
Future-Focused	Past or Present Focused
Receives More Resources	Receives Fewer Resources
External Threat	Internal Threat
Perceived High Level of Threat to Economy	Perceived Low Level of Threat to Economy
More Perceived Risks	Fewer Perceived Risks
Fewer Real Risks	More Real Risks
Patients Viewed as Victims	Patients Viewed as Offenders
Focus on Public Health Interventions	Focus on Self-Management
Reactive and Proactive Efforts	Primarily Reactive Efforts
Profitable to Treat	Profitable to Manage

**Figure 1.**
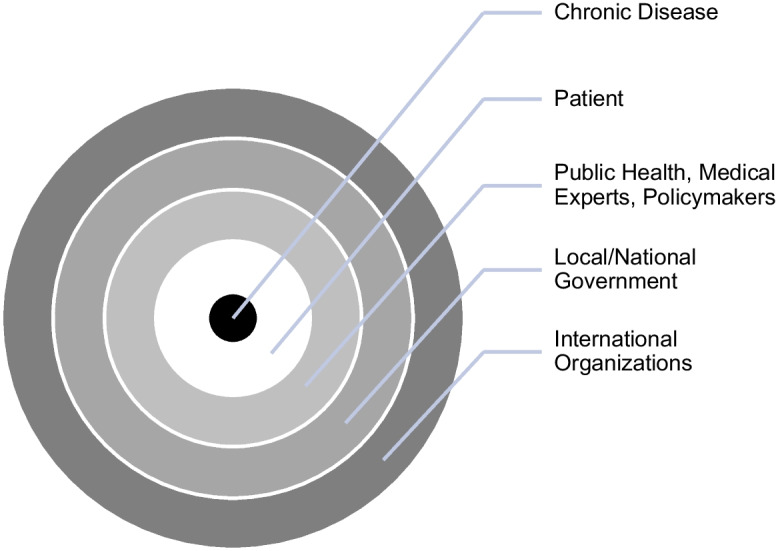
Perceived Relationship Towards Chronic Disease (Diabetes)

**Figure 2:**
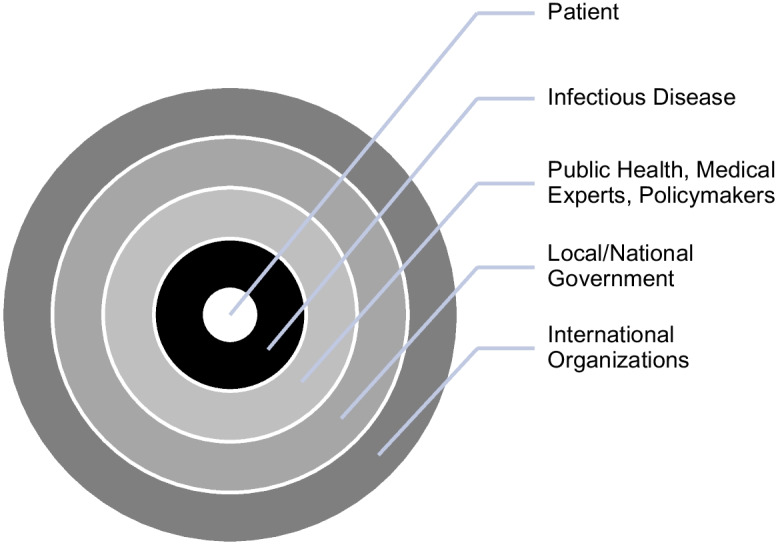
Perceived Relationship Towards Infectious Disease (COVID-19)

Large coordination of efforts was called for and required during the COVID-19 pandemic to lessen the impact of disease on human health and the health of the global economy. Similar large-scale coordination across sectors on all levels of local and national interdisciplinary efforts, however, has not been conducted in regard to diabetes with the urgency that it demands. Below is the framework of infectious (COVID-19) and chronic diseases (diabetes) as presented by the case studies reviewed (figures 1 and 2). This framework is helpful in visually illustrating the relationship between disease and different stakeholders. It is especially helpful to visualize the relationship between disease and the patient based on the perceptions of risk and responsibility. The perceived relationship towards chronic diseases such as T2D (figure 1) illustrates that the individual (patient) stands between the disease and the rest of the stakeholders. The efforts of public health and medical experts, policymakers, local governments, and international organizations are aimed at helping individuals govern disease by self-regulation. The chronic disease itself is visually presented to be inside the individual; thus, the individual holds the primary responsibility for the disease.

The perceived relationship towards infectious diseases such as COVID-19 (figure 2) illustrates that the disease stands between the individual (patient) and the rest of the stakeholders. The infectious disease itself is visually presented to be outside the individual. This is in line with the perception of COVID-19 as an external environmental threat. Thus, different stakeholders hold the primary responsibility for the disease, which requires a shared response to a common threat. The efforts of public health and medical experts, policymakers, local governments, and international organizations are aimed at the disease to govern it through increased public health interventions.

The visual representation of disease in relation to different stakeholders highlights that the relationship between chronic and infectious diseases, as exemplified by COVID-19 and diabetes, differs. It differs in terms of who holds the responsibility for the health condition and how it should be best approached. It also demonstrates where the patient stands in the relationship to disease and those in the position of authority. Efforts to manage and regulate human bodies and lives to achieve health are aimed through a number of levels, as illustrated, towards the centre. When looking at chronic diseases, these efforts are aimed at the patient, who is then expected to conform to established norms, medical expertise, and disease management guidelines to change behaviour and habits. When looking at infectious disease, collective responsibility to address disease calls for public health interventions, stockpiling, and increased preparedness efforts. The difference in prioritization efforts conforms with such perception of disease. This perception helps clarify that to achieve a better state of health in the future, individuals suffering from chronic disease must take responsibility for their health today, better manage their health, and ensure that the choices they make are in line with regulatory expert recommendations and norms. To achieve a better state of health in the future for individuals suffering from infectious diseases, the state and global health organizations must be better prepared to handle the spread of infectious diseases.

**Figure 3:**
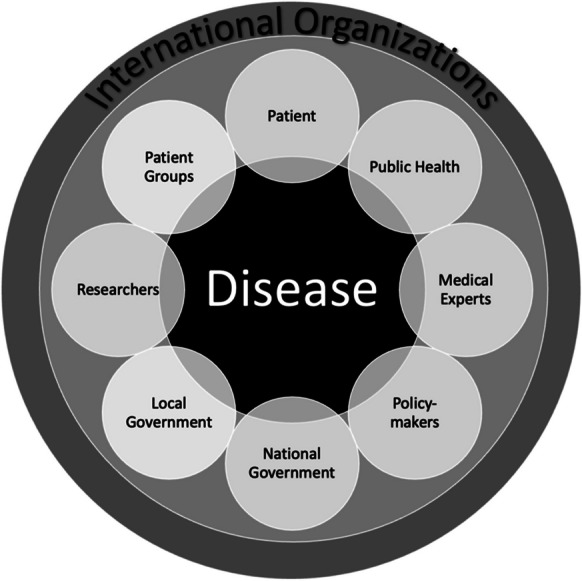
Reframed Relationship Towards Disease

The figures above offer a dualistic view of disease categories, but health conditions do not fall into neat categories with clear distinctions. They often overlap in complex ways with infectious diseases leading to chronic illness and chronic diseases having an acute onset with important implications both for individual’s well-being and for the economy. One way to reframe the relationship towards disease is illustrated in figure 3 below. It places disease and the human suffering from illness on equal footing regardless of disease categorization. Whether we are considering chronic and infectious diseases or even a chronic infectious disease, such as HIV/AIDS, it is important to reconceptualize human health as equally important and equally valued across levels, stakeholders, and populations. Health as a human right is not a new idea (WHO 2023d), but it is an important one. It necessitates a collective response to disease regardless of its category, onset, origins, or perceived risks. It requires collective responsibility for the reduction of human suffering that cannot be easily quantified.

The ethics of choice in national and global health prioritization points to the need to recognize human suffering beyond the economic drivers and address it in a more equitable way across levels and populations. The mortality information and the scale of human suffering it represents should be used to improve how people live without obscuring or interfering with our understanding of how the disease impacts human life. A more equitable approach to human health is to recognize disease as a threat regardless of its type. The suggested reframing of the relationship towards disease shifts the focus of biopower toward a more holistic approach without prerequisites or constraints. Similar approaches have been suggested to recognize and account for the complexity of interactions around disease, including the One Health Approach (CDC 2023c), the Patient-Centric Approach (Brantly and Brantly 2020), and the Whole Health Approach (Gaudet and Kligler 2019). The proposed framework distributes the authority over health not only among the local or national governments, policymakers, public health, medical experts, or even researchers (academic or commercial) but also among patient and patient groups. This allows for all stakeholders to take responsibility for the disease without shifting blame. Disease as a common adversary requires collaboration across sectors and across levels of governance. Ultimately, disease, whether it is chronic or infectious, is a cause of suffering and poses significant threats to human life and livelihood.

A disease is both a pathological reality and a social construction, and disease management is not only clinical but also cultural (Hays 1997). It is not sufficient to rely on data and technologies to address the complexity of human conditions. Nor is it sufficient to take on the perspective of a few over many different stakeholders when addressing human health. The visualization of the relationship towards disease provides insights into the politics over life across levels, from individual to population, and across focus areas. It helps explain that the skewed perception of disease skews national and global public health priorities. When it comes to disease, people are both offenders and victims since there is always something one can do to improve health, and there are always outside factors posing a threat. Efforts to control, manage, and reshape individuals through technological means are influenced by political authorities in alliance with pervasive industry involvement. Reframing disease considerations requires all to shift the frame of thinking not only pertaining to risk but also pertaining to responsibility for disease states.

## Conclusion

Knowing why people die does not determine how we approach people’s lives today. Human mortality rates, other health statistics, and the changing disease trends do not align with U.S. and global efforts to improve how people live. The ideas of human health and disease are entangled with the public perception of risk and responsibility. These perceptions are influenced by national and global efforts to optimize life to ensure better health for all in the future using profitable data and technologies. Quantification of health and the increased use of technologies are some of the primary tools used to ensure the “ideal” state of health across populations. Risk and responsibility-based understanding of disease influences efforts to address illness in the name of security and better health outcomes in the future. Disease and the discrepancies in national and global health priority setting can also be framed as a biopolitical problem.

Despite the blurred boundaries of disease categories or the synergistic interactions among diseases, the assignment of responsibility for one’s health treads the ambiguously traced outline of chronic and infectious diseases. This work helped frame the perceived relationship towards disease to visually illustrate the ways that interactions among the individuals, disease, and other stakeholders are conceptualized. The lens of biopolitics was particularly helpful in illuminating the discrepancy in global health priority setting. It shows that biopolitics at the nexus of infectious and chronic disease skews national and global public health priorities rooted in the ways we perceive and understand humans and disease. This work also proposed an alternative framework for a more equitable approach to understanding disease and the relationship between human health, impacted individuals, the community of regulatory experts, and those in positions of authority.

Diseases and their categorization present complex entanglements. They require further study to include the investigations of pandemic discourses and states of emergency from the perspective of risk production and ethical decision-making. Nations collect data regarding human mortality, yet this data is not reflected accurately in the efforts undertaken and the responsibilities assigned to improve how people live. The COVID-19 pandemic has emphasised that no one is safe from disease without action at all levels. Effective national and global health programmes are effective only with national and global commitment to an improved understanding of the relationship between disease, the patient, and society.

## Data Availability

Not applicable.
